# Has Rift Valley fever virus evolved with increasing severity in human populations in East Africa?

**DOI:** 10.1038/emi.2016.57

**Published:** 2016-06-22

**Authors:** Marycelin Baba, Daniel K Masiga, Rosemary Sang, Jandouwe Villinger

**Affiliations:** 1 International Centre of Insect Physiology and Ecology (ICIPE), Nairobi 00100, Kenya; 2 Department of Medical Laboratory Science, PMB 1069, University of Maiduguri, Maiduguri 600230, Nigeria

**Keywords:** animals, East Africa, epizootic, humans, outbreak, Rift Valley fever

## Abstract

Rift Valley fever (RVF) outbreaks have occurred across eastern Africa from 1912 to 2010 approximately every 4–15 years, most of which have not been accompanied by significant epidemics in human populations. However, human epidemics during RVF outbreaks in eastern Africa have involved 478 deaths in 1998, 1107 reported cases with 350 deaths from 2006 to 2007 and 1174 cases with 241 deaths in 2008. We review the history of RVF outbreaks in eastern Africa to identify the epidemiological factors that could have influenced its increasing severity in humans. Diverse ecological factors influence outbreak frequency, whereas virus evolution has a greater impact on its virulence in hosts. Several factors could have influenced the lack of information on RVF in humans during earlier outbreaks, but the explosive nature of human RVF epidemics in recent years mirrors the evolutionary trend of the virus. Comparisons between isolates from different outbreaks have revealed an accumulation of genetic mutations and genomic reassortments that have diversified RVF virus genomes over several decades. The threat to humans posed by the diversified RVF virus strains increases the potential public health and socioeconomic impacts of future outbreaks. Understanding the shifting RVF epidemiology as determined by its evolution is key to developing new strategies for outbreak mitigation and prevention of future human RVF casualties.

## Introduction

**Figure 1:**
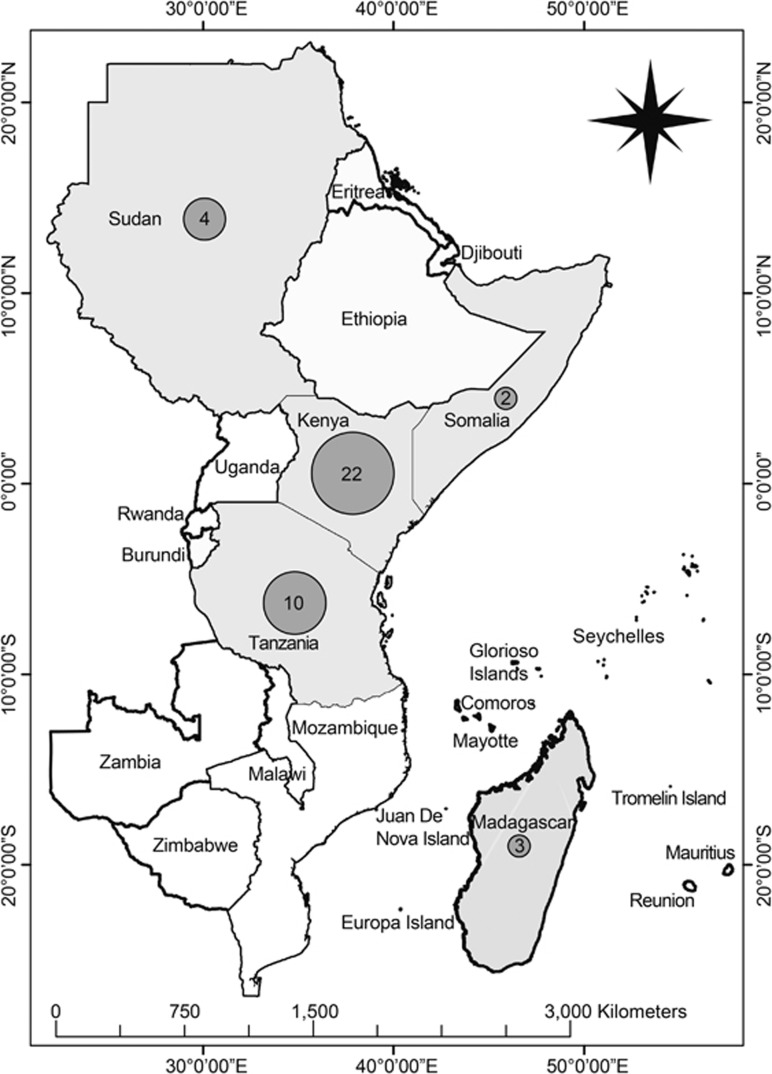
Map of eastern African countries indicating frequencies of major RVF outbreaks over the past century (1912–2010). The numbers of outbreaks in specific countries are indicated in the gray spheres.

Rift Valley fever (RVF), caused by the Rift Valley fever virus (RVFV; genus: *Phlebovirus*, family: *Bunyaviridae*), is an arboviral disease primarily of domesticated animals that also causes mild to life-threatening disease in humans. The virus is a negative-sense, single-stranded RNA virus, and its name is derived from the Great Rift Valley of Kenya, where the disease was first recognized and characterized in 1912^1^ and first described in 1931 after a highly fatal epizootic in Kenya in 1930.^2^ Subsequently, RVF epizootics/epidemics have occurred (Figure 1) in 4–15-year cycles in association with flooding above the normal rainfall in many flood-prone habitats.^3^

RVFV is inherently complex, posing a significant challenge to outbreak prediction, mitigation and management.^4^ Characteristically, once RVFV is introduced into permissive ecologies, the virus becomes endemic/enzootic, making the region vulnerable to periodic outbreaks with the potential to spread further into non-endemic environments that have favorable conditions.^5, 6^ For example, in Tanzania, the number of villages affected by RVF outbreaks increased from two in 1930 to 175 in 2006 and 2007, and the number of cases increased 11-fold from 1026 in 1930 to 11 339 in 1978 and another 12-fold to 136 750 in 2007 in 21/28 districts.^5^ Similarly, in Kenya, RVF outbreaks were limited to 1/69 administrative districts from 1912 to 1950, but in 2006 and 2007, 38/69 districts were affected.^6^ Furthermore, in both Kenya and Tanzania, the districts that formed the primary cluster of the 1977–1978 and 1997–1998 outbreaks became part of the relatively larger cluster of outbreaks in 2006/2007.^5, 6^ As the virus causes severe outbreaks with large-scale livestock losses and, more recently, significant impacts on human health, it poses an emerging zoonotic threat to vulnerable African communities. Although the virus is endemic to sub-Saharan Africa (SSA), it has the potential for global spread, as it has already crossed significant natural geographic barriers such as the Indian Ocean, the Sahara Desert and the Red Sea to reach naive ecologies.^5^

As a zoonotic disease, RVF disproportionately affects vulnerable communities with poor resilience to economic and environmental challenges through its wider societal effects. The RVFV has been associated with congenital abnormalities (stillbirths, mummified fetuses, defects of the central nervous system, musculoskeletal problems, hydranencephaly, hydroencephaly, porencephaly, arthrogryposis and cerebellar hypoplasia) in fetal or neonatal ruminants.^7^ During the outbreaks in Africa, vertical transmission of RVFV has been reported in mosquito vectors, ruminants and, most recently, humans.^8^ This clearly reflects the burden of this emerging zoonotic infectious disease to humans, especially among women and pregnant women.^9^

Despite its enormous public health and socioeconomic impacts, RVF is often neglected by major global donors and disease control programs.^4^ However, in recent years, the classification of RVFV as a potential bio-/agro-terrorism agent has triggered global interest, particularly in the area of vaccine development and diagnostics.^4^ We review the history of RVF outbreaks in East Africa and the epidemiological risk factors that could have influenced RVF epidemiology in past outbreaks. In the context of the persistent and intensifying epidemics in humans, we specifically examine the genetic evolution of RVFV as well as the socioeconomic impacts of these outbreaks in East Africa.

## History

In the first six decades after the viral disease was described (1931–1996), human RVF cases during livestock RVF outbreaks in East Africa were rare and generally mild.^10^ Although the first human death due to RVF was reported in 1934, this was in a laboratory worker who was investigating RVF cultures,^11^ presumably because of exposure to very high titers. Widespread epizootics in the region were not accompanied by similar epidemics of the disease in human populations for >50 years.^10^

Of the 23 RVF outbreaks in Kenya between 1912 and 2007 (Table 1), 14 affected 3–38 of the 69 districts nationally, whereas nine were localized to 1–2 of the 69 districts.^6^ Twenty of both the national and localized RVF outbreaks in Kenya (1912–1989) scarcely involved human populations.^25^ For instance, RVF epizootics from 1950 to 1951 in Kenya resulted in the deaths of an estimated 100 000 sheep, but without significant impact on human health.^5^ Thereafter, RVF epizootics re-occurred in 1961 and 1964, and from 1989 to 1991 in farms that were <20 km from the site of the 1931 outbreak.^5^ These outbreaks primarily involved domestic sheep and cattle, especially those imported to Kenya.^10^ During the 1989 epizootics, 12 of the 30 herdsmen with detectable RVFV IgG (four with RVFV-specific IgM) had handled the affected animals,^10^ but neither experienced clinical disease typical of RVF nor could recall any illness during the outbreak period. A previous report revealed low levels of RVFV antibody in human populations,^26^ despite several reported cases of epizootics in East Africa in the early 1900s.^2, 27^ In addition, the RVF epizootic in 1989 in Kenya occurred at a farm and involved 80%–90% of the cattle, but only two out of 26 herdsmen had detectable RVF antigen and had no clinical illness.^10^

Similarly, Tanzania has experienced nine RVF epizootics between 1930 and 1997 without significant morbidity and mortality in humans.^3, 5^ However, in Sudan, RVF epizootics that resulted in 100% morbidity and 40% mortality in livestock did coincide with human cases in 1973 and 1976.^13^ However, these human cases were limited to two laboratory staff who had contact with the infected animals and developed a mild disease that resolved spontaneously within two weeks with neither complication nor death.^13^ In Sudan, RVF was also implicated in a 1988–1989 outbreak of acute febrile illness that was primarily attributed to Sandfly fever Naples virus, a *Phlebovirus* that is closely related to RVF. However, this assessment was based on the fact that 26% of the 185 patients had RVFV IgG antibodies,^28^ which are indicative of past RVF infections and cannot be used to identify causative agents of current infections.

Since the first major RVF outbreak in humans in 1977 in Egypt,^29^ there have been several RVF outbreaks with significant impact on human health in eastern Africa. The 1997–1998 RVF outbreaks, which occurred in Kenya, Somalia and Tanzania simultaneously, resulted in an estimated 89 000 human infections and 478 deaths in Kenya and Somalia, but no human involvement was reported in Tanzania.^3, 5, 16, 17^ These estimates were based on clinical features such as acute onset of fever and headache associated with hemorrhage (hematochezia, hematemesis and bleeding from other mucosal sites), but specimen from fatal cases were not available. However, 47% (17/36) of the blood samples from other ill patients who were tested at the National Institute of Virology, South Africa, and the Center for Disease Control and Prevention, USA, had acute RVFV infections detected by IgM antibodies, virus isolation, reverse-transcriptase PCR for viral nucleic acid or immunohistochemistry.^16^ From 2006 to 2007, RVF outbreaks re-occurred with significant mortality in humans (Table 1) in Kenya,^18, 19, 20, 30^ Somalia^20^ and Tanzania,^5, 29^ and eventually extended to Sudan in 2007 and 2008,^21, 22^ and Madagascar in 2008.^23^ RVF outbreaks re-occurred in Sudan in 2010, affecting both animals (abortion in ewes and does) and humans with a history of contact with aborted fetal material,^9, 24^ but little information is available about these outbreaks. The RVF epidemiology during the 2007–2008 outbreaks in eastern Africa changed from being originally associated with livestock to considerably infecting humans and resulting in high fatality rates.^9^ As the human RVF cases identified in more recent outbreaks have been more severe in terms of their associated morbidity and mortality, we postulate that insufficient surveillance and case finding, lack of diagnostic capabilities, poor health infrastructure, under-recognition, underreporting and underestimation by medical personnel and inadequate public awareness may not necessarily be the major determinants of the insignificant impact of RVF on human populations reported in East Africa in the early 1900s.

Human RVF outbreaks in other African countries in the 1900s were not significantly more extensive than what occurred in East Africa. In South Africa, symptomatic human RVF infection started in 1950 and 1951 with one case (no death) and in 1974 and 1975 with 110 cases (1 death),^31^ but Mauritania and Senegal reported 220 deaths in 1987^32^ and 300–400 cases (six deaths) in 1997 and 1998.^33^ In Madagascar, the first and less severe RVF epizootic occurred in 1979 and re-occurred from 1990 to 1991^15^ with a significant loss of animals, but no human involvement. However, another RVF epizootic in Madagascar in 2008 involved 476 human cases and 19 deaths (case fatality rate (CFR)=4%).^23^ Interestingly, human involvement in the affected countries was initially limited to those who had contact with infected animals,^34^ but was later extended with increasing severity to those who could not recall any contact with any animals in the subsequent epidemics.^35^

In 1977 and 1978, RVF outbreaks occurred in Egypt, resulting in 20 000–200 000 estimated human cases with 600 deaths.^9, 29^ In Saudi Arabia and Yemen, RVF outbreaks in 2000 involved 2171 human infections and 245 deaths.^36^ It has been suggested that the RVF outbreaks in Saudi Arabia and Egypt were imported from Kenya and Sudan, respectively, through infected animals. The report that the same RVF virus strain was implicated in the 1997–1998 RVF outbreaks in Kenya as in the 2000 outbreaks in Saudi Arabia and Yemen^36^ seems to correlate with the 89 000 estimated human infections (478 deaths) in Kenya.^5, 16, 17^ However, it is unclear how the same virus that caused only mild illness in humans with no complications in Sudan was implicated with a high incidence rate of human infection and death (75 000 estimated with 698 confirmed infections and 222 deaths) after it was imported to Egypt.^9^ Notably, the 1989 RVF epizootics in Kenya were confirmed by virus isolation, plaque reduction neutralization tests and indirect fluorescent antibody tests, whereas earlier confirmatory tests were less stringent.^12^ Although each country in East Africa now has a coordinated inter- and intra-sectoral outbreak response and disease mitigation strategies in place, the enormous negative impacts of each RVF outbreak demands new and systematic approaches to preventing or reducing the resultant outcomes if and when outbreaks do recur. An improved prediction algorithm with a lead period of >six months as well as integrated and sustainable approaches between different governmental sectors and organizations within and between countries are necessary to holistically address future RVF outbreaks.

## Epidemiological risk factors associated with past RVF outbreaks in East Africa

### Ecological factors

Unlike the majority of arboviruses that adapt to a narrow range of vectors, the RVFV infects a wide range of vectors including mosquitoes (with *Aedes* and *Culex* as the major vectors), flies and ticks.^37^ Interestingly, different species of vectors have different roles in sustaining the transmission of RVFV in an environment.^38^ Usually, flooded dambos (low-lying areas of soil) in East Africa induce the hatching of transovarially infected eggs of *Aedes* mosquitoes that are dormant in the soil, which serve as primary vectors (eggs can remain viable for several decades).^39^ Hatched infectious mosquitoes transmit the virus to nearby livestock and wildlife vertebrate hosts, which serve as amplifiers of the virus, infecting more mosquitoes, and thereafter secondary vectors of the virus (*Culex, Anopheles* and *Mansonia* mosquitoes) amplify the transmission of the virus to non-infected domestic animals and humans.^40^ Flooding in areas with a high density of livestock and/or wildlife creates a conducive environment for RVF transmission,^18^ and under such conditions, the virus is maintained within the ecosystem. Other environmental factors such as canopy cover, dissolved oxygen, pH, turbidity, organic matter, salinity and temperature also influence the abundance of various mosquito vector species and arbovirus transmission.^41^

RVF epidemiology in East Africa is closely associated with the ecological factors prevalent in the Great Rift Valley (a long depression in the earth that runs down the eastern side of Africa), which traverses Ethiopia and Kenya to northern Tanzania with two branches that form the eastern and western drainage ecosystems. The western branch runs through Tanzania and Uganda, and the eastern branch runs through Kenya and Tanzania.^42^ Interestingly, the ten explosive RVF outbreaks in Tanzania between 1930 and 2007 occurred in the eastern wing of the Great Rift Valley of northern Tanzania.^5^ The clay and loamy soil texture of the Rift Valley support long periods of water retention (flooding) and render it suitable for breeding primary mosquito vectors and the survival of their RVFV-infected eggs.^5^ A significant association has been observed between RVF outbreaks from 1930 to 2007 and the clay and loamy soil textures in the eastern Rift Valley ecosystem of Tanzania, where clustering of RVF outbreaks has persistently and predominantly been detected.^3, 22^ Unlike sandy soil, clay soil texture supports the retention of water for long periods of time, and thereby contributes to the flooding and wetness of the habitat, making it suitable for the breeding and survival of mosquito vectors.^5, 9^

### Climatic factors

Climate determines the geographic and temporal distribution and the life cycles of arthropod vectors as well as the dispersion and evolution of associated arboviruses. It also defines the efficiency with which arboviruses are transmitted from arthropods to vertebrate hosts.^43^ Climatic variables indirectly affect vector abundance and distribution, and their ability to vector arboviral diseases.^44^ One vector species may be displaced by another with a different vectorial capacity in response to environmental changes, such as deforestation, expansion in irrigation or increase in brackish water breeding sites due to rises in sea level.^45^

Historical outbreaks in East Africa have been linked to periods of abnormally high rainfall with a few localized exceptions, such as the 1989 Kenyan outbreak that was related to local heavy rainfall at the focus of the outbreak.^10^ Such conditions depend on El Niño/Southern Oscillation (ENSO)-related climate anomalies that are based on a combination of satellite measurements of elevated sea-surface temperatures and satellite-derived normalized difference vegetation index data.^46^ These data were used to retrospectively predict RVF outbreaks in Kenya between 1950 and 1998,^47^ and prospectively predicted the 2006–2007 RVF outbreaks in the Horn of Africa with 2–6-month lead times.^47^ The association of RVF outbreaks with ENSO anomalies, which are increasing with climate change,^48^ also has implications for the duration of future inter-epidemic periods (IEPs).

During past RVF outbreaks in East Africa, excessive rainfall that averaged 1720 mm (as experienced during El Niño years) in Kenya, Somalia and Tanzania led to flooding and increased vegetation cover that favored a high vector density and species diversity.^14^ The flooding caused several lakes, rivers and dambos in the epicenters of RVF outbreaks to overflow.^49^ Notably, a large proportion of RVFV, which remain dormant within the eggs of mosquitoes in the soil for several years, are hatched during flooding, resulting in intensified transmission of the virus to herds and human hosts within the environment during these outbreaks. However, the absence of RVF outbreaks during some IEPs in Kenya (ranging from one to seven years), despite heavy rainfall and flooding, indicates that other unknown drivers of RVF may exist in vulnerable ecologies. Nonetheless, IEPs may signal the time required for herd immunity to fall to levels permissive for the spread of the virus.^5, 9^ Therefore, there is a need to definitively define all of the drivers of RVF outbreaks in vulnerable ecologies if future RVF outbreaks are to be abated.

The impact of rainfall on the presence, absence, size and persistence of breeding sites depends upon the local evaporation rates, soil type, slope of terrain and the proximity of large bodies of water (e.g., rivers, lakes and ponds), whereas wind has a significant effect on vector distribution.^50^ Overall, high relative humidity favors most metabolic processes in vectors towards their prolonged survival, whereas low humidity tends to decrease their daily survival rate due to dehydration and dessication.^50^ In some cases, low humidity and high temperatures accelerate the metabolic rate of a vector, increasing biting rates and frequency of blood feeding (in an attempt to compensate for high levels of water loss), which lead to enhanced egg production and increases in vector populations.^50^ However, extremely high temperatures may be detrimental to vector populations.^50^ Consequently, the geographical range or distribution of vectors tends to be limited by a minimum and maximum temperature/humidity. Although there is no clearly defined pattern of average annual maximum or minimum temperatures associated with RVF outbreaks, they have shown a tendency to cease as the maximum and minimum monthly temperatures decline.^5^ Moreover, higher extrinsic incubation temperatures of RVF mosquito vector eggs (*Aedes taeniorhynchus* and *Culex pipiens*) have been shown to result in earlier RVFV dissemination and transmission,^51^ which should increase their replication, transmission and evolutionary rates. We therefore speculate that global climate change may select for adaptive changes in the RVFV that may also influence its host range, virulence, pathogenicity and/or transmission efficacy.

### Human behavioral factors

Land use changes such as deforestation, irrigation for farming, application of fertilizer in farms and the building of residential houses are strongly linked to the emergence and re-emergence of arboviral disease.^52^ The majority of the RVF epizootics from 2006 to 2007 was livestock in pastoral and agro-pastoral farming systems.^16, 53^ In addition, one of the RVF outbreaks in Sudan was linked to the construction of the dam of Merowe on the Nile River basin, as it provided new breeding sites for RVF vectors.^22^ The inability of mosquitoes to fly more than a few hundred meters during their lifetime may limit their role in long-range disease dissemination.^54^ However, uncontrolled livestock, human movement and/or the importation of animals can contribute to the spatiotemporal spread of RVF outbreaks from endemic to naive areas.^5^ Generally, human RVF cases are uncommon in the absence of animal disease occurrence.^5^ Nevertheless, through serosurveys, inter-epidemic human RVFV transmission has been reported in East Africa in the absence of reported or observed outbreaks.^55^ However, because these studies were restricted to human populations, it is difficult to ascertain that an animal infection did not precede human exposure in the studied environment.

RVFV has been detected in both livestock^56, 57^ and waterbuck during IEPs.^6^ As wildlife, which has been considered to be a possible reservoir of RVF, live in close proximity to livestock, they may contribute to the amplification of the virus during epizootics.^6^ Human RVF is significantly associated with direct contact with tissue, tissue fluids and mucous membranes of infected animals or animal products as well as with infectious fomites, small droplets and mosquito bites. Generally, RVFV infects humans by either inoculation (parenteral route) through a wound from contaminated surgical instruments or contact with broken skin or inhalation of aerosols produced during the slaughter or birthing of infected animals.^21^ Previous studies have revealed that direct contact or handling of infected animals and being a herdsperson or veterinarian were significantly associated with acute RVFV infection in humans.^16, 30, 34^ Touching an aborted animal fetus was found to be associated with severe RVF disease (hemorrhage, encephalitis or ocular disease), possibly due to an exposure to high quantities of RVFV through aerosolization of the virus when handling a carcass.^16, 58^ This speculation is supported by a report that showed that laboratory workers acquired RVF through aerosol transmission.^55^ In another report, consuming or handling products from sick animals was significantly associated with severe disease and death.^30^ However, no study has shown that RVFV is transmitted through the oral–fecal route. Therefore, ingestion of uncooked infected animals or unpasteurized milk may not necessarily drive transmission, as previously reported.^30, 34^ Nonetheless, as consuming or handling products from sick animals has been found to be significantly associated with acute RVF infection, severe illness and death,^17^ inhalation of infectious aerosols in the process of preparing, handling and consuming these uncooked infected animals or their products may be the major mode of transmission of RVFV to humans. Indeed, aerosol exposure to RVFV causes earlier and more severe neuropathology in the mice.^59^ The transmission of RVFV to humans through mosquito bites is limited, but it may be very difficult to quantify because the bites may have gone unnoticed.^14^

Other human behavioral factors that favor RVF spread during outbreaks include poor inter-ministerial collaboration and inadequate surveillance of livestock and human populations due to limited resources.^36, 58^ The severity of RVF epidemics has been exacerbated by delays in recognizing the risk factors and in making timely decisions to prevent and control the disease.^60^ In addition, insufficient data on the herd immunity level in both animal and human populations, and the lack of entomological surveillance to identify risk-prone areas in some vulnerable ecologies^61^ may contribute to frequent RVF outbreaks.

The delayed implementation of outbreak responses and other disease mitigation strategies tends to exacerbate the impacts of a disease on lives, livelihoods and local, national and regional economies. Due to limited resources, the inability of veterinary authorities and vaccine manufacturers to maintain stocks of vaccine, which have short shelf-lives and could expire before they are used or sold, has contributed to the spread of the disease and the associated socioeconomic impact.^3^ Although the existing livestock vaccine^62^ has a shelf-life of ~4 years, the IEPs between major outbreaks can be up to 10 years or even 20 years.^3^ However, the procurement of vaccines when epidemics/epizootics have already started could be time-consuming, allowing the disease to spread in the meantime. The production of effective livestock vaccines that have long shelf-lives to conform to the episodic nature of RVF has become a necessity. The prediction algorithm that successfully predicted the 2006–2007 RVF outbreaks in Africa^47^ resulted in a lead period that was not long enough for a sustainable and effective implementation of disease mitigation strategies.^47^

## Genetic evolution of RVFV

The widespread epizootics of RVF among ruminants in the early twentieth century were not accompanied by similar epidemics in human populations.^14^ The evolutionary rate of RVFV has diversified an ancestral virus that existed 120–130 years ago into multiple extant RVFV strains.^63^ Therefore, the past 100+ years since RVFV was first identified have been sufficient for major evolutionary changes to occur through mutation and genetic reassortment. However, low genetic diversity of the virus was reported during the 1977, 1983 and 2006–2007 epidemics in Egypt, Mauritania^63^ and East Africa,^40^ respectively. According to Bird *et al.*,^63^ RVFV genetic diversity primarily involves the accumulation of mutations at an average of 2.9 × 10^−4^ substitutions per site per year, with some evidence of RNA segment reassortment.

The apparent stability of the RVFV genome has been attributed to high vertical transmission rates and the negative selection pressure exerted by diverse alternating vertebrate hosts and arthropod vectors. However, the low nucleotide diversity observed may be due to the limited diversification time from a recent common ancestor rather than the stability of the genome,^64^ especially considering the low replication rates during IEPs when large proportions of the virus are dormant in mosquito eggs. Indeed, the genetic diversity of RVFV isolates has computationally coalesced to a single putative ancestral sequence from ~1880–1890,^65^ only 40–50 years before RVF was first identified in 1931.^2^ However, purifying negative selection may lead to an underestimation of the age of viral lineages.^66^ Because RVFV can persist in dormant eggs for long periods of time, outbreaks in endemic areas are associated with an intensified transmission of multiple lineages, albeit with low divergence^40, 64^ and larger epidemic disease incidence.^66^ In contrast, RVF outbreaks in naive ecologies (Egypt in 1977, Mauritania in 1983, and Saudi Arabia and Yemen in 2000) have been associated with newly introduced single lineages of RVFV with minimal genetic diversity^63^ through animal movement and mosquito vectors. A mathematical model to assess the spread of RVF revealed that a lack of herd immunity allowed the virus to expand geographically, leading to longer transmission intensification periods and delayed infection rates before it became an epidemic, thus involving many dispersed vulnerable ecologies.^65^

A possible instability of the RVFV genome has been demonstrated by a report showing that the glycoprotein (Gn) (associated with virus attachment and entry into cells) sequences of the isolates involved in the 2006–2007 outbreak had as high as 2.2% amino-acid substitutions compared with isolates from the outbreaks between 1944 and 2002.^40^ Six of these amino-acid substitutions represented distinct mutations from all known historical viruses, and one of these, a glycine-to-arginine substitution at position 216 of the Gn protein, was found only in isolates from the virulent human infections.^40^ The emergence of potentially more virulent and pathogenic virus genotypes can occur within short time frames, resulting from the of only a few key amino acids.^40^ This speculation is supported by reports of other arboviruses in which amino-acid substitutions significantly have been shown to alter their behavior and virulence in recent decades. In the USA, neuroinvasive and non-virulent strains of West Nile virus differ by five amino acids in their envelope proteins.^67^ Similarly, a single nucleotide change in the envelope protein (E1-A226V) of the Chikungunya virus^68^ has been associated, in part, with increased fitness in a new vector species (*Aedes albopictus*) in a region that lacked the typical *Aedes aegypti* vector, as well as with viral infectivity and dissemination and transmission rates.^69^ Thus, any slight change in the genetic constitution of an arbovirus should not be ignored. Indeed, a single amino-acid change in a virus genome can act through multiple phenotypic effects to create an epidemic.^70^ Therefore, we speculate that the relatively small changes in RVFV genomes and the accumulation of these changes over several decades could have influenced its virulence (as estimated by the incidence and CFRs), host preferences, vector competence and transmission rates. Such changes could facilitate its establishment in new ecologies following its introduction through animal movement and adaptation to available vector populations.

In addition, the evolution of RVFV strains may be enhanced by its segmented genome's propensity to undergo segment reassortment in nature. Partial sequences of all three RNA segments have shown evidence of reassortment in SSA RVFV isolates.^71^ The convergence of some lineages within genome segments implies that reassortment has played an evolutionary role in the history of RVFV.^40^ Co-infection with different RVFV strains may facilitate the formation of reassortant RVFV chimera that may proliferate in the environment as they are taken up and transmitted by vectors.^72^ A reassortant RVFV was reported to differ from all other isolates during the 1977 RVF outbreak in Egypt, but it showed similarities with strains that caused human deaths.^64^ Interestingly, such reassortment has also been hypothesized to be responsible for the increased virulence of RVF in humans in an original report.^29^ If attenuated RVF vaccines with unattenuated segments are used for vaccination during outbreaks, the chances of co-infection between wild and vaccine strains may be increased. Consequently, the diversification of RVFV genotypes through reassortment into chimeric viruses with unpredictable levels of virulence could be accelerated. It is therefore important to use vaccines in which all three segments are attenuated^73^ and/or are replication deficient.^74^ In addition, limiting the vaccination of livestock to during IEPs could decrease the chances of co-infection between wild and vaccine strains of RVFV.

**Figure 2:**
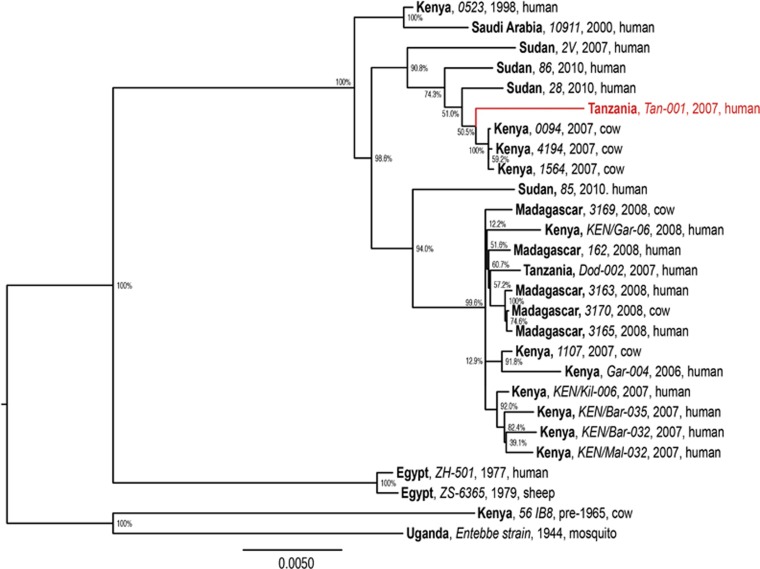
Maximum likelihood phylogenetic tree of complete RVFV genome sequences (combined L (GenBank accessions: DQ375400–1, DQ375406, DQ375410, DQ375427, DQ375429, EU574004, EU574017, EU574020, EU574029, HM586953–60, JF311371, JF311375–6 and JQ820488–91), M (GenBank accessions: DQ380190–1, DQ380196–7, DQ380200, DQ380205, JQ820488–91, EU574031, EU574044, EU574047, EU574055, HM586964–71, JF311368–9, JF311371, JF311375–6 and JQ820483–6) and S segments (GenBank accessions: DQ380145, DQ380149, DQ380156, DQ380169–70, DQ380176, EU574057, EU574086, EU574072, EU574075, HM586975–82, JF311386–7, JF311389, JF311393–4, JQ820472, JQ820474, JQ820476 and JQ820477)) analyzed using PhyML v. 3.0.^75^ The phylogenies employed the General Time-Reversible nucleotide substitution (rate categories=4) model, in which the base frequencies and the relative substitution rates between them were estimated by maximizing the likelihood of the phylogeny. For estimating the tree topology, both nearest-neighbor interchange and sub-tree pruning and regrafting improvements were used. Country (bold), isolate identification (italics), year and host are indicated for all 27 sequences analyzed. Bootstrap values at the major nodes are expressed as percentage agreement among 1000 replicates. The branch length scale represents substitutions per site. A red sequence indicates a reassortant isolate.

**Figure 3:**
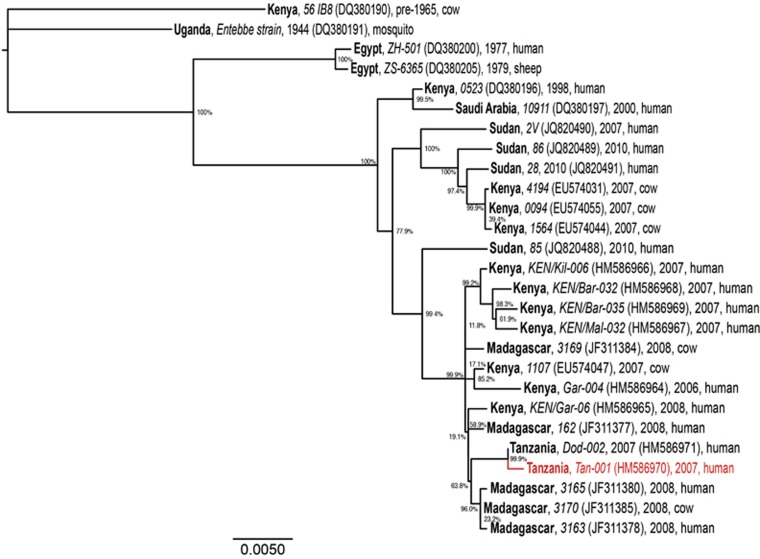
Maximum likelihood phylogenetic tree of complete RVFV M segment sequences. Country (bold), isolate identification (italics), GenBank accession (in brackets), year and host are indicated for all 27 sequences analyzed. Bootstrap values at the major nodes are expressed as percentage agreement among 1000 replicates. The branch length scale represents substitutions per site. A red sequence indicates a reassortant isolate.

The molecular phylogeny of RVFV genomes (Figure 2) demonstrates the diversification of distinct RVFV lineages within East Africa, especially within the past decade, during which RVF outbreaks have had a greater impact on human health. We found that the genomic phylogeny (Figure 2) corresponds closely with those of the S and L segments (not shown); however, the M segment phylogeny (Figure 3) reveals clear reassortment of the Tanzanian *Tan-001* isolate from 2007, for which only the M segment clusters with the other Tanzanian isolate (*Dod-002*) among isolates from Madagascar. The isolate's L and S segments, as shown in the full genome phylogeny (Figure 2), cluster among the isolates from Kenya and Sudan. In contrast, all three segments of the *Dod-002* isolate cluster among isolates from Madagascar. Reassortant RVFVs have also been reported among Kenyan isolates^63^ and with increased pathogenicity in humans in South Africa.^64^ The Madagascar isolate 3169 might also be a reassortant, as its full genome is most closely related to other Madagascar RVF viruses (Figure 2), whereas its M segment clusters among Kenyan isolates (Figure 3). This latter case is much more subtle and inconclusive, but it shows that reassortment may be occurring more frequently among more closely related RVF genomes than can be identified phylogenetically. The diversification of RVFV genomes through mutation and assortment mechanisms could have generated lineages that are virulent in humans,^40^ some of which may potentially induce abortion and neonatal mortality in humans.^76^ Although ecological factors and human behaviors may impact the frequency and location of its occurrence, genetic factors significantly impact the virulence of the virus as well as the disease severity in its hosts.

## Socioeconomic impacts of RVF outbreaks in East Africa

The increase in human mortality and morbidity associated with RVF outbreaks in the past two decades has exacerbated both their social and economic impacts. Although human RVF mortality was low (CFR <1%) during the twentieth century, mortality rates rose to as high as 23%–47% during the East African 2006 and 2007 outbreaks, possibly due to the genetic evolution of the virus. Even for RVF survivors, the neurological and visual complications are likely to be lifelong, with a considerable economic impact due to a loss in disability-adjusted life years (DALYs) estimated to range between 353–11 958 and 188–6530 for 2005.^77^ These DALY estimates for RVF were deduced from the possibly lifelong neurological and visual complications/blindness that caused substantial economic impact in RVF survivors.^55^ Hospitalization due to arboviral diseases results in both monetary loss and loss of time, with a significant impact of deaths on families, communities and countries in the region. Similarly, the psychological distress caused by RVF on the affected families is unquantifiable, and the loss of a productive member of the family in terms of labor, income and parental care results in serious consequences. Those who were seriously affected by the outbreak claimed that RVF represented a worse threat to health than the commonly dreaded human immunodeficiency virus/acquired immune deficiency syndrome.^14^

RVF is known to induce abortions and perinatal mortality (>95%) in herds used for meat, dairy production and income generation, leading to reduced food availability and income. These effects are more severe on poorer dwellers who lack alternative sources of livelihood.^23^ In Tanzania, the loss of livestock during the 2006–2007 RVF outbreak was estimated to be US $ 4 243 250 for cattle and US $ 2 202 467 for goats and sheep.^30^ The imposition of trade bans for three years on the exportation of live animals from the Horn of Africa exacerbated the impact of RVF-induced losses. In Tanzania, the export of cattle dropped by 54% during the 2006–2007 RVF outbreak, and the domestic livestock market flow dropped by 37%. In Kenya, the loss was estimated at over US $32 million.^78^ The overall economic loss during the 2006–2007 RVF outbreaks in East Africa was estimated to exceed $60 million.^79^ Such enormous losses are catastrophic to low-income and impoverished countries, as the governments of the affected countries are compelled to mobilize funds from different sources to contain the outbreak. In addition, the loss of earnings from the livestock trade results in a lack of finance for basic amenities such as education, health, food and shelter.^80^ Furthermore, movement bans on animals have caused animal traders to incur additional costs by keeping the animals they purchased before the bans were enforced.^14^ The associated loss of income for herd owners has damaged the pride, prestige, integrity and self-importance of livestock owners among their peers. Livestock traders were forced to rely on their past savings to such an extent that they lacked the financial capital to resume trading activities, even when the outbreak was contained.^14^ Overall, the political, psychological and economic implications of RVF may have contributed to an intentional underreporting of the symptoms, thus confounding the estimates of the size and impact of outbreaks.

## Conclusions

Since its discovery in 1912 and the 1930s, RVF has undergone continued geographic expansion through East Africa with increasing human disease. Persistent and episodic outbreaks of RVF caused by different lineages of the virus that localize in particular areas have occurred in several eastern African countries. The endemic areas of RVF may periodically experience outbreaks and serve as the index foci for future outbreaks in non-endemic environments. The epidemiology of past RVF outbreaks demonstrates a complex interaction between environmental, viral and host factors. The RVFV that primarily caused the epizootics in East Africa in the twenty-first century has evolved from an ancestral virus that existed 100–200 years ago into multiple extant strains that have caused increased disease severity in humans. The evolution of RVFV through mutation and reassortment and the accumulation of these changes over several decades may have changed the disease epidemiology, increasing its geographic distribution and severity in human populations. Therefore, understanding RVF epidemiology and its clinical symptoms in humans, as determined by its evolution, is an essential step in developing new strategies for outbreak mitigation and prevention of future human RVF casualties. The enormous human health and socioeconomic impacts, including basic food security, that are imposed by RVF make it imperative to adopt more stringent and effective measures to mitigate its impacts.

## Figures and Tables

**Table 1:** RVF outbreaks in eastern Africa, 1912–2010

**Outbreaks**	**Year**	**Country**	**District/province**	**Diagnostic test used**	**Estimated number of cases in human**	**Number of confirmed cases in humans (deaths)**^a^	**Reference**
Epizootic (localized)	1912	Kenya	Nakuru	Unknown	Unknown	None	^6^
Epizootic (localized)	1915	Kenya	Nakuru	Unknown	Unknown	None	^6^
Epizootic (national)	1930	Kenya	Nakuru, Naivasha, Marura, Ndabibi, Ol Magogo and Njoro	Virus isolation	None	None	^2^
Epizootic (national)	1930, 1947, 1957, 1960, 1963, 1968	Tanzania	Unknown	Unknown	Unknown	None	^5^
Epizootic (national)	1936	Kenya	Nakuru, Naivasha, Marura, Ndabibi, Ol Magogo and Njoro	Virus isolation	None	None	^6^
Epizootic (national)	1951	Kenya	Nakuru, Trans Nzoia, Uasin Gishu and Laikipia districts of Rift Valley Province; Thika, Nyeri and Maragwa districts of Central Province; Nairobi Metropolitan district of Nairobi Province	Unknown	None	None	^3^
Epizootic (national)	1955	Kenya	Nakuru, Trans Nzoia, Uasin Gishu and Laikipia districts of Rift Valley Province; Thika, Nyeri and Maragwa districts of Central Province; Nairobi Metropolitan district of Nairobi Province	Unknown	None	None	^6^
Epizootic (national)	1960–1961	Kenya	Nakuru, Trans Nzoia, Uasin Gishu, Laikipia, Narok, Kajiado, and West Pokot districts of Rift Valley Province; Thika, Nyeri, Maragwa and Kiambu districts of Central Province; Nairobi Metropolitan district of Nairobi Province; Garissa, Wajir, and Mandera districts of Northeastern Province; Isiolo, Marsabit, Machakos, and Makueni districts of Eastern Province; Kwale, Kilifi, and Tana River districts of Coast Province	Unknown	None	None	^6^
Epizootic (national)	1964	Kenya	Nakuru, Trans Nzoia, Uasin Gishu, Laikipia, Narok, Kajiado and West Pokot districts of Rift Valley Province; Thika, Nyeri, Maragwa and Kiambu districts of Central Province; Nairobi Metropolitan district of Nairobi Province; Garissa, Wajir and Mandera districts of Northeastern Province; Isiolo, Marsabit, Machakos and Makueni districts of Eastern Province; Kwale, Kilifi and Tana River districts of Coast Province	Unknown	None	None	^6^
Epizootic (localized)	1965	Kenya	2 of the previously affected districts (not specified)	Unknown	None	None	^6^
Epizootic (national)	1967–1968	Kenya	12 of the previously affected districts (not specified)	Virus isolation and serology	None	None	^6, 12^
Epizootic (localized)	1969	Kenya	2 of the previously affected districts (not specified)	Unknown	None	None	^6^
Epizootic (national)	1970–1971	Kenya	3 of the previously affected districts (not specified)	Unknown	None	None	^6^
Epizootic/epidemic	1973, 1976	Sudan	Nile, Khartoum, Kassala, El Gezira, Sennar and White Nile districts in Northern Province	Virus isolation	Unknown	2 (0)	^13^
Epizootic (national)	1977–1978	Kenya	11 of the above districts (not specified)	Unknown	None	None	^6^
Epizootic	1977–1978	Tanzania	Northern part	Unknown	Unknown	None	^14^
Epizootic	1979	Madagascar	Antananarivo	Unknown	None	None	^12^
Epizootic (national)	1981	Kenya	8 of the previously affected districts (not specified)	Unknown	None	None	^6^
Epizootic (national)	1983	Kenya	9 of the previously affected districts (not specified)	Unknown	None	None	^6^
Epizootic (localized)	1985–1986	Kenya	1 of the previously affected districts (not specified)	Unknown	None	None	^6^
Epizootic (localized)	1987–1988	Kenya	1 of the previously affected districts (not specified)	Unknown	None	None	^6^
Epizootic (national)	1988–1989	Tanzania	Northern part	Unknown	Unknown	None	^14^
Epizootic (national)	1989–1991	Kenya	9 of the previously affected districts (not specified)	Virus isolation and serology	None	None	^12^
Epizootic	1990–1991	Madagascar	Antananarivo	Unknown	None	None	^15^
Epizootic/epidemic (national)	1997–1998	Kenya, Somalia and Tanzania	22 of the 27 previously affected districts (not specified)	Virus isolation, RT-PCR and serology	89 000	0 (478)	^3, 5, 16, 17^
Epizootic (localized)	1999	Kenya	1 of the previously affected districts (not specified)	Unknown	None	None	^6^
Epizootic (localized)	2002	Kenya	1 of the previously affected districts (not specified)	Unknown	None	None	^6^
Epizootic/epidemic (national)	2006–2007	Kenya	Previously affected districts and Baringo, Samburu, Kirinyaga, Murang'a, Taita-Taveta, Lamu, Malindi, Kitui, Meru (Central and north), Mwingi, Moyale, Embu, and Mbeere districts	Virus isolation, RT-PCR and Serology	75 000	700 (158), CFR=22.6%	^18, 19, 20^
Epizootic/epidemic (national)	2006–2007	Somalia	Lower Juba Gedo Hiran, Middle Juba, Middle Shabelle, Lower Shabelle regions.	Unknown	30 000	114 (51), CFR=45%	^20^
Epizootic/epidemic (national)	2006–2007	Tanzania	Arusha, Dares Salaam, Dodoma, Iringa, Manyara, Morogoro, Mwanza, Pwani, Singida, Tanga regions.	Unknown	40 000	309 (144), CFR=46.6%	^20^
Epizootic/epidemic (national)	2007–2008	Sudan	White Nile, Sennar, El-Gazira, Sennar (near White Nile and Blue Nile Rivers) River Nile, Khartounm and Kassala States	Serology	75 000	747 (230), CRF=30.8%	^21, 22^
Epizootic/epidemic (national)	2008	Madagascar	Alaotra Mangoro, Analamanga, Itasy, Vakinakaratra and Anosy Regions.	Unknown	Unknown	476 (19), CFR=4.1%	^23^
Epizootic/epidemic	2010	Sudan	El Gezira State	Unknown	Unknown	Unknown	^24^

Abbreviation: case fatality rate, CFR.

a CFR was calculated only with confirmed cases.
